# JAK2-Positive Myeloproliferative Neoplasm: Polycythaemia Vera Presenting As Budd-Chiari Syndrome

**DOI:** 10.7759/cureus.115000

**Published:** 2026-08-22

**Authors:** Ramangouda Malipatil, Srinidhi Rao V R, Nagaraja B S, Mumtaz Ali Khan

**Affiliations:** 1 Internal Medicine, Bangalore Medical College and Research Institute, Bangalore, IND

**Keywords:** budd-chiari syndrome, jak2 v617f mutation, myeloproliferative neoplasm, polycythemia vera, splanchnic vein thrombosis

## Abstract

Budd-Chiari syndrome (BCS), caused by hepatic venous outflow obstruction, is a critical diagnosis often masking an underlying myeloproliferative neoplasm (MPN). We present the case of a 33-year-old female whose diagnosis was driven by hepatic decompensation rather than typical haematological symptoms of MPN. Initial imaging confirmed the anatomical pathology, revealing chronic BCS on computed tomography (non-visualised hepatic veins, hepatic vein stenosis, and classic caudate lobe hypertrophy). The subsequent work-up noted a paradoxical haematological picture (significant neutrophilic leucocytosis (up to 55,570 cells/mm³) and relative thrombocytosis) despite splenomegaly and portal hypertension. This finding, combined with the CT evidence, prompted definitive genetic screening, which confirmed the diagnosis of an MPN by detecting the JAK2 exon 14 c.1849G>T (p.Val617Phe) mutation (variant allele frequency of 79.2%). This case underscores the importance of utilising radiological evidence in the initial stages and recognising the combination of an atypical blood picture alongside hepatic decompensation as a key indicator for an underlying MPN, allowing for prompt molecular diagnosis and targeted therapy.

## Introduction

Splanchnic vein thrombosis (SVT) comprises thrombotic obstruction of the portal, mesenteric, splenic, or hepatic veins and represents a clinically significant form of venous thromboembolism because of its strong association with systemic disorders, particularly myeloproliferative neoplasms (MPNs). Although uncommon, with an estimated incidence of 0.7-2.7 per 100,000 person-years, SVT often presents a diagnostic challenge because classical haematologic manifestations of MPNs may be absent or obscured by hypersplenism and haemodilution associated with portal hypertension and chronic liver disease [1,2].

Among the SVT subtypes, portal vein thrombosis (PVT) is the most frequently encountered, with an estimated incidence of two to four per 100,000 person-years [3]. A meta-analysis by Smalberg et al. demonstrated that the mean prevalence of underlying MPNs and JAK2 V617F positivity in PVT was 31.5% and 27.7%, respectively [4]. Furthermore, routine JAK2 V617F testing identified occult MPNs in 15.4% of patients without overt haematologic abnormalities, highlighting the value of molecular screening even in apparently idiopathic cases [4].

Budd-Chiari syndrome (BCS), characterised by hepatic venous outflow obstruction, is considerably rarer, with an annual incidence of approximately one per million individuals [5]. However, its association with MPNs is even stronger than that observed in PVT. In the same meta-analysis, the prevalence of MPNs and JAK2 V617F positivity in patients with BCS was 40.9% and 41.1%, respectively, with polycythaemia vera (PV) occurring significantly more frequently in BCS than in PVT [4]. Notably, JAK2 V617F testing identified previously unrecognised MPNs in 17.1% of patients with BCS despite the absence of classical haematologic features [4].

These findings underscore the importance of routinely evaluating patients with unexplained SVT for an underlying MPN. Accordingly, current international guidelines recommend JAK2 V617F mutation testing in all patients presenting with unexplained SVT, irrespective of their haematologic profile [5]. We report a case of a young woman presenting with chronic BCS and hepatic decompensation, in whom molecular testing revealed JAK2-positive PV despite an atypical haematologic and biochemical profile, emphasising the importance of maintaining a high index of suspicion for occult MPNs in patients with extensive splanchnic venous thrombosis.

## Case presentation

A 33-year-old female presented with progressive abdominal distension and bilateral lower limb swelling for two months, with marked worsening over the preceding week. The symptoms were insidious in onset and gradually progressive, with no identifiable aggravating or relieving factors. There was no history of altered sensorium, gastrointestinal bleeding, fever, cardiac symptoms, or features suggestive of connective tissue disease. She reported occasional abdominal pain that was relieved with medications. She had experienced an episode of jaundice three years earlier that resolved with medical treatment; however, ascites had developed during the same period, following which she experienced intermittent episodes of abdominal distension. There was no significant family history.

On general examination, bilateral pedal oedema was present. Abdominal examination revealed gross abdominal distension with dilated veins over the abdominal wall, positive shifting dullness, consistent with gross ascites, and splenomegaly. Based on the clinical presentation, a provisional diagnosis of decompensated chronic liver disease with portal hypertension was considered.

**Figure 1:**
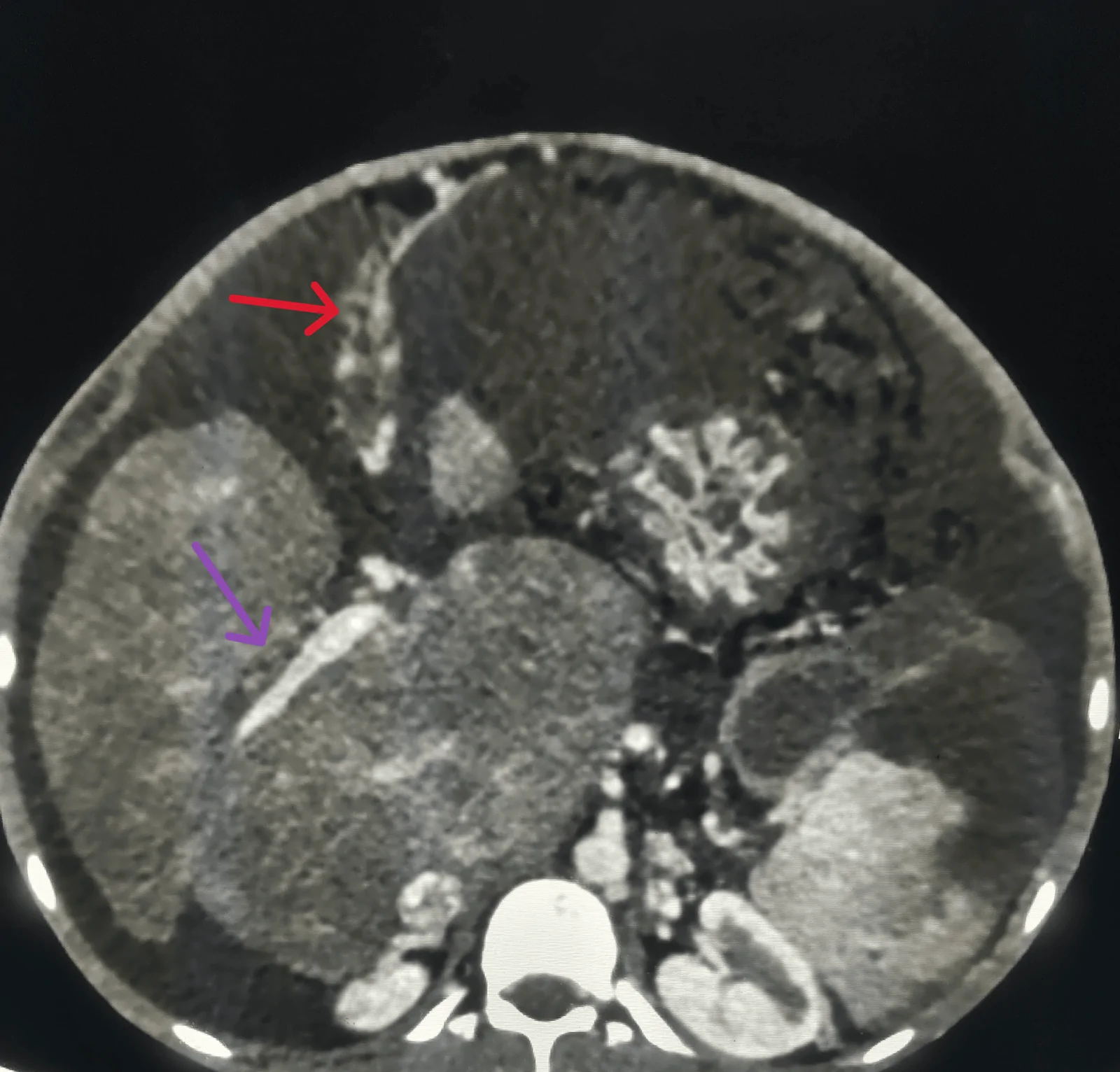
Axial contrast-enhanced CT section of the upper abdomen (hepatic region) showing recanalisation of the paraumbilical vein (red arrow) and visualisation of only a single portal vein branch (purple arrow), consistent with portal hypertension.

**Figure 2:**
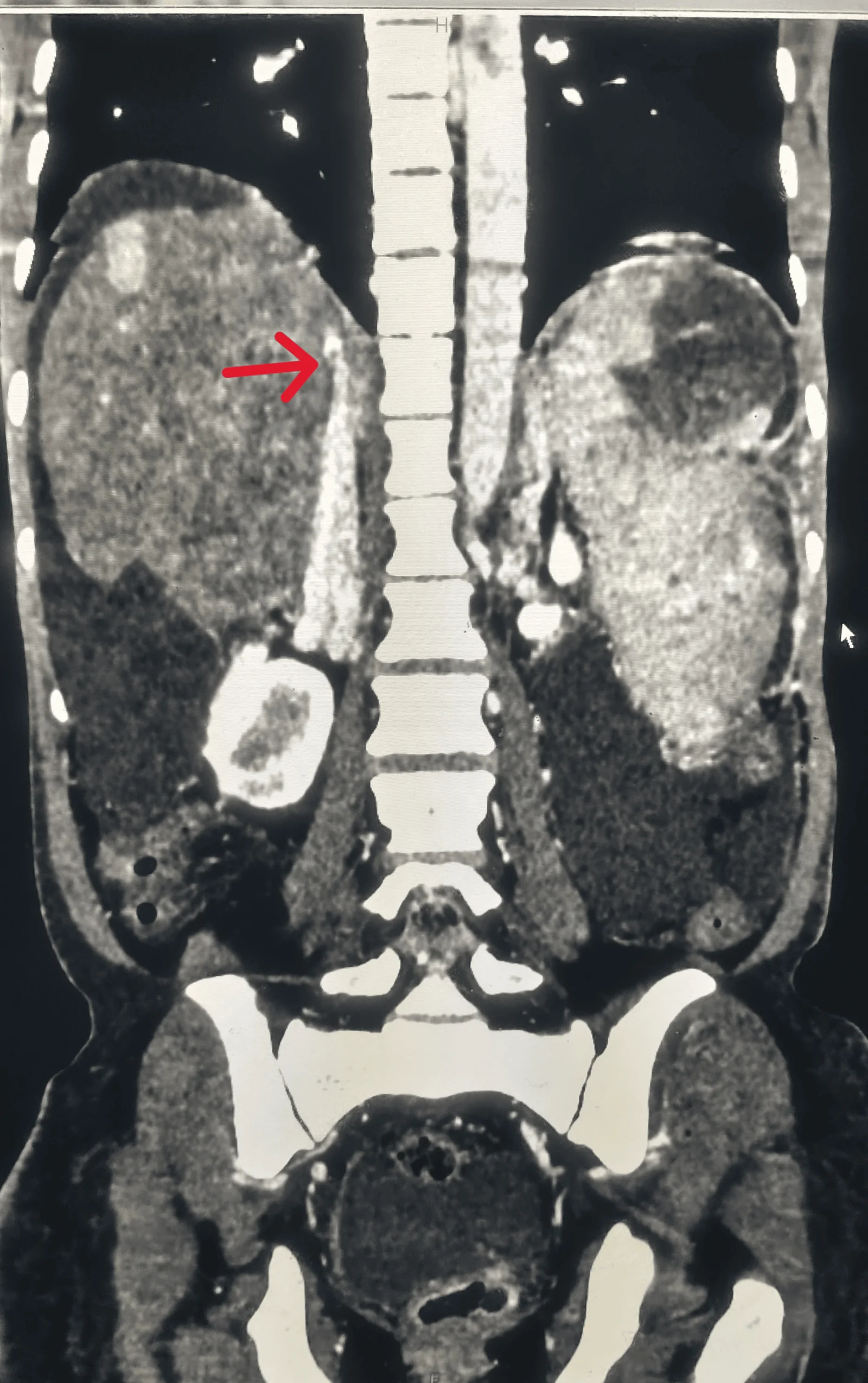
Coronal contrast-enhanced CT section of the upper abdomen demonstrating an attenuated intrahepatic inferior vena cava (red arrow) with non-visualisation of the hepatic veins, consistent with hepatic venous outflow obstruction.

**Figure 3:**
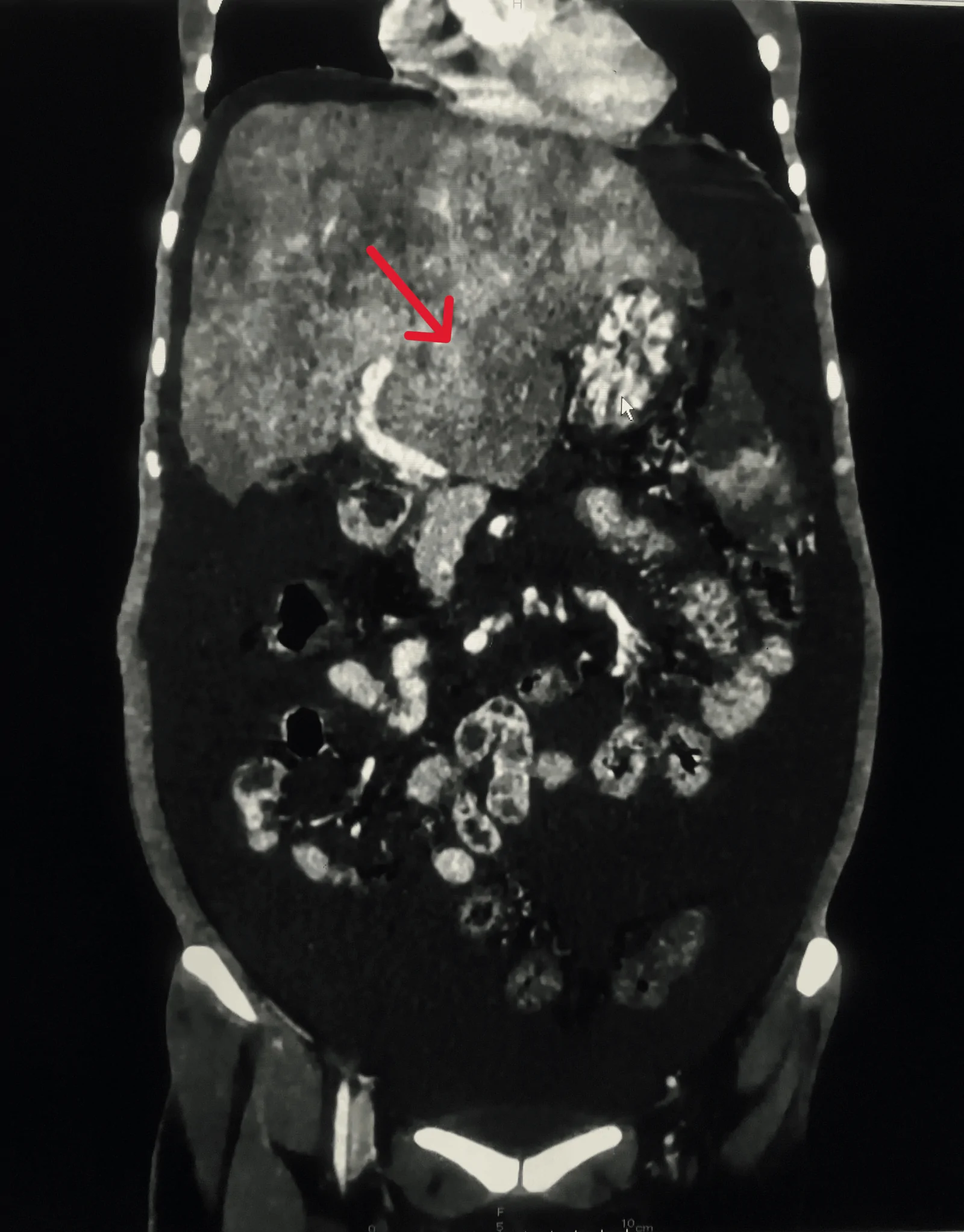
Coronal contrast-enhanced CT section of the upper abdomen demonstrating hypertrophy of the caudate lobe of the liver (red arrow), a characteristic radiological feature of Budd-Chiari syndrome.

Contrast-enhanced computed tomography (CT) of the abdomen demonstrated a recanalised paraumbilical vein with visualisation of only a single portal vein branch, suggesting significant portal hypertension (Figure 1). The CT also showed an attenuated intrahepatic inferior vena cava with non-visualisation of the hepatic veins, findings consistent with hepatic venous outflow obstruction (Figure 2). In addition, hypertrophy of the caudate lobe was noted, a characteristic radiological feature of BCS (Figure 3).

The laboratory investigations are summarised in Table 1. The patient had marked neutrophilic leucocytosis (55,570/mm³), normal haemoglobin (15.2 g/dL), and a normal platelet count despite significant portal hypertension (376 × 10³/mm³). Liver function tests showed hypoalbuminaemia, while renal function was normal. Ascitic fluid analysis revealed a high serum-ascites albumin gradient (SAAG) with low protein content, reflecting portal hypertension in advanced chronic BCS, and no evidence of spontaneous bacterial peritonitis, malignancy, or tuberculosis.

**Table 1:** Laboratory and ancillary investigations HCV: hepatitis C virus; HIV: human immunodeficiency virus; INR: international normalised ratio; MCHC: mean corpuscular haemoglobin concentration; MCV: mean corpuscular volume; PCV: packed cell volume; PMN: polymorphonuclear neutrophil; PT: prothrombin time; RBC: red blood cell; VAF: variant allele frequency; WBC: white blood cell

Investigation	Day 1	Day 6	Two weeks after hydroxyurea initiation	Reference range
Haemoglobin	15.2 g/dL	15.89 g/dL	9.57 g/dL	Female: 12.0-15.5 g/dL
Total WBC count	55,570/mm³	39,980/mm³	400/mm^3^	4,000-11,000/mm³
Neutrophils	83%	92%	3.41%	40-75%
Lymphocytes	6.8%	4.27%	93.96%	20-45%
Monocytes	7.2%	-	-	2-10%
Eosinophils	2.9%	-	-	1-6%
Basophils	0%	-	-	0-1%
PCV	48.9%	51%	-	36-46%
MCV	69.4 fL	70.3 fL	24 fL	80-100 fL
MCH	31 g/dL	31.2 g/dL	31 g/dL	32-36 g/dL
Platelet count	376,000/mm³	385,000/ mm³	54000/mm^3^	150,000-450,000/mm³
Reticulocyte count	3.1%	-	-	0.5-2.5%
Peripheral smear	Normocytic normocytic RBCs; marked neutrophilic leukocytosis with left shift; large platelets; no blasts	-	-	-
Renal function tests				
Blood urea	55.8 mg/dL	-	116.9 mg/dL	15-40 mg/dL
Serum creatinine	1.01 mg/dL	-	1.39 mg/dL	0.6-1.1 mg/dL
	Liver function tests			
Total bilirubin	0.97 mg/dL	-	2.72 mg/dL	0.3-1.2 mg/dL
Direct bilirubin	0.70 mg/dL	-	2.60 mg/dL	<0.3 mg/dL
AST	40.4 U/L	-	32 U/L	10-40 U/L
ALT	38.6 U/L	-	16 U/L	7-35 U/L
ALP	144 U/L	-	95 U/L	44-147 U/L
Total protein	5.18 g/dL	-	3.3 g/dL	6.0-8.3 g/dL
Albumin	3.05 g/dL	-	1.7 g/dL	3.5-5.0 g/dL
A/G ratio	1.43	-	1.06	1.0-2.2
Electrolytes				
Sodium	127 mmol/L	-	132 mmol/L	135-145 mmol/L
Potassium	5.18 mmol/L	-	5.5 mmol/L	3.5-5.0 mmol/L
Chloride	86 mmol/L	-	97 mmol/L	98-106 mmol/L
Coagulation profile				
PT	11 seconds	-	-	11-14 seconds
INR	1.06	-	-	0.8-1.2
	Ascitic fluid analysis			
Total cell count	80 cells/mm³	-	-	-
Polymorphs/lymphocytes	60/40%	-	-	-
Protein	0.5 g/dL	-	-	<2.5 g/dL (low protein ascites)
Albumin	0.47 g/dL	-	-	SAAG > 1.1
Cytology for atypical cells	Negative	-	-	-
ADA	2.1 U/L	-	-	-
Serology				
HBsAg	Non-reactive	-	-	Negative
Anti-HCV	Non-reactive	-	-	Negative
HIV	Non-reactive	-	-	Negative
Autoimmune work-up				
ANA	Negative	-	-	Negative
Autoimmune hepatitis panel	Negative	-	-	Negative
Prothrombotic work-up				
Protein C	66% (likely secondary depletion)	-	-	83-168%
Protein S	Normal	-	-	60-130%
Lupus anticoagulant	Negative	-	-	Negative
Anticardiolipin antibodies	Negative	-	-	Negative
Homocysteine	11.6 µmol/L	-	-	5-15 µmol/L
Factor V Leiden mutation	Not detected	-	-	Not detected
Bone marrow biopsy	The biopsy showed hypercellular marrow with normoblastic erythropoiesis, a relative increase in the myeloid series, and marked megakaryocytic proliferation with prominent clustering. Megakaryocytes demonstrated morphological abnormalities including bulbous/hypolobulated nuclei and micromegakaryocytes. The original report described the findings as “hypercellular marrow with increased megakaryopoiesis showing clustering,” with further evaluation recommended for an underlying myeloproliferative/myelodysplastic neoplasm.	-	-	-
Serum erythropoietin	23.3 mIU/mL	-	-	2.5-18.5 mIU/mL
Molecular studies	JAK2 V617F mutation detected (VAF 79.2%); negative CALR and MPL	-	-	-

An extensive evaluation to identify the underlying cause of BCS was performed (Table 1). Viral hepatitis markers, HIV serology, autoimmune work-up, antiphospholipid antibodies, lupus anticoagulant, homocysteine levels, and factor V Leiden mutation were negative. Protein C levels were mildly reduced, likely reflecting secondary depletion due to liver disease, whereas protein S levels were normal.

Given the marked leucocytosis and an upper-normal platelet count, an MPN was suspected. Bone marrow biopsy demonstrated a hypercellular marrow with prominent megakaryocytic clustering. However, histopathology images were unavailable. Molecular analysis detected a JAK2 V617F mutation with a variant allele frequency of 79.2%, while CALR and MPL mutations were absent. Interestingly, the serum erythropoietin (EPO) level was at the upper limit of the normal reference range, an atypical finding in JAK2-positive MPNs. Based on the clinical, radiological, histopathological, and molecular findings, the patient was diagnosed with BCS secondary to JAK2-positive MPN, resulting in decompensated liver disease.

**Figure 4:**
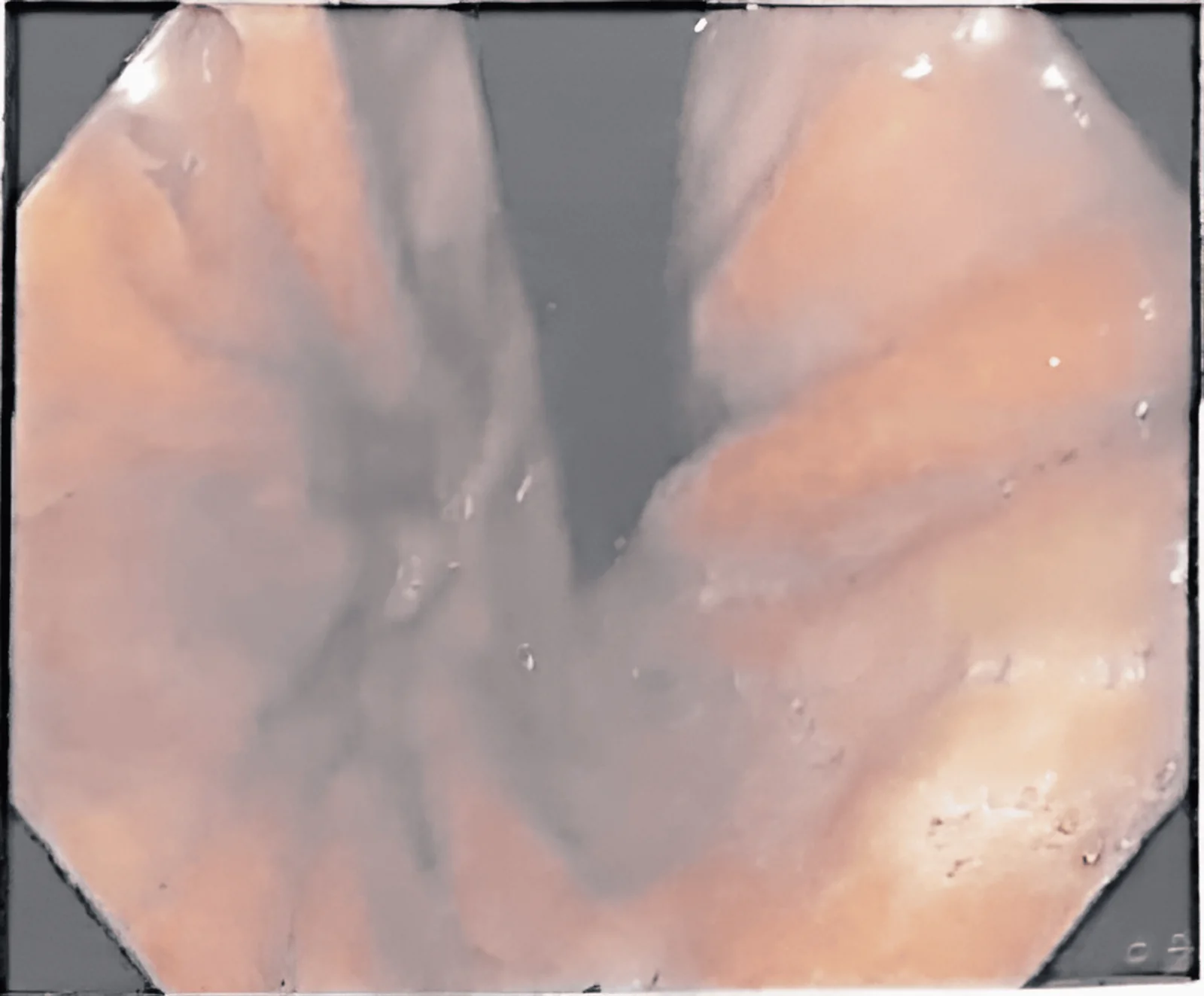
Upper gastrointestinal endoscopy demonstrating five columns of large oesophageal varices. Endoscopic variceal ligation was performed with deployment of five bands.

**Figure 5:**
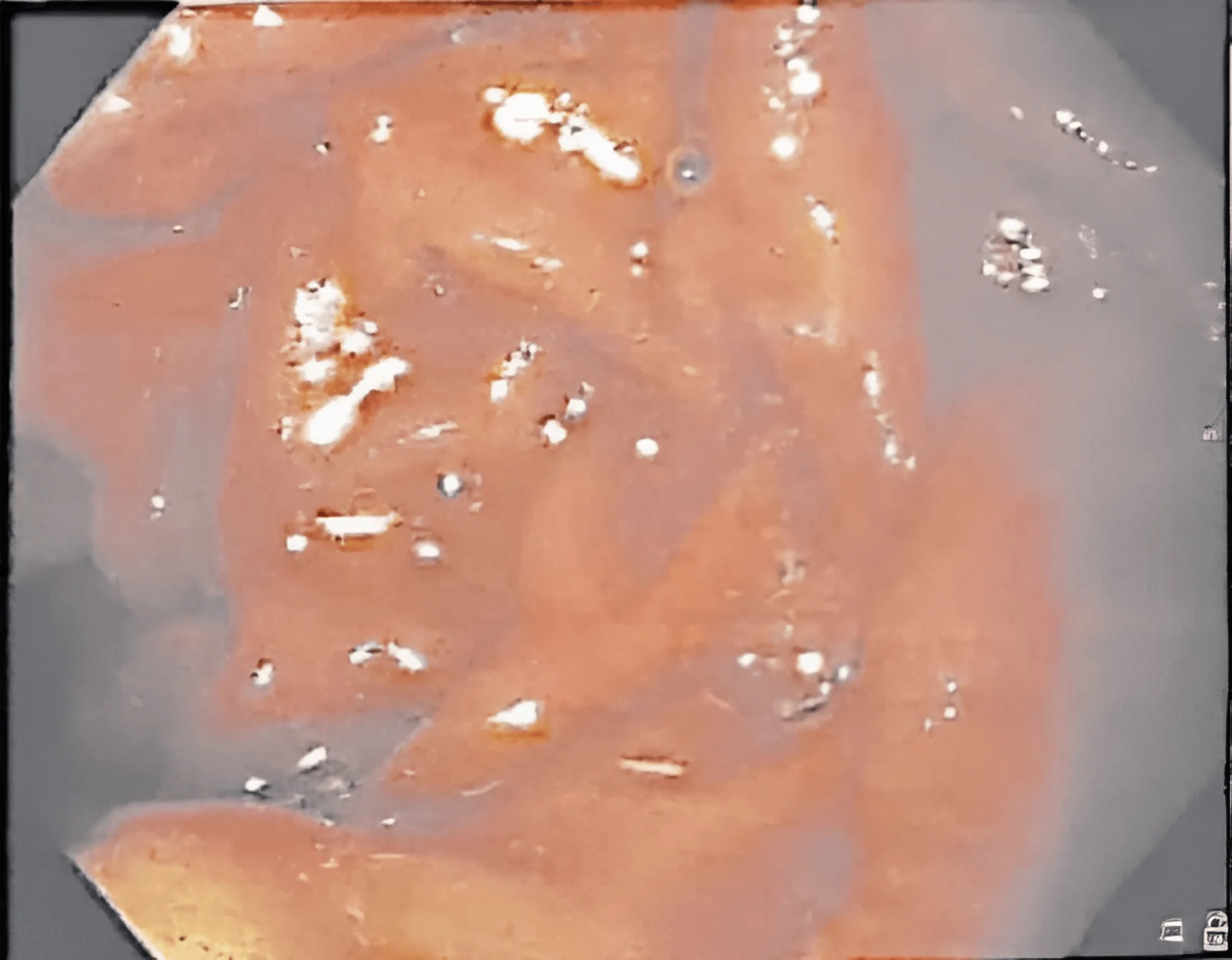
Upper gastrointestinal endoscopy demonstrating portal hypertensive gastropathy with a mosaic mucosal pattern involving the fundus, body, and antrum. No fundal varices were identified.

The patient was initially managed with therapeutic paracentesis with albumin for tense ascites, along with diuretics (spironolactone and furosemide), salt restriction, and supportive care. Long-term anticoagulation was initiated with low molecular weight heparin (LMWH) followed by warfarin (vitamin K antagonist). Cytoreductive therapy with hydroxyurea (500 mg twice daily) and aspirin (75 mg once daily) was started following confirmation of the underlying MPN. Upper gastrointestinal endoscopy demonstrated five columns of large oesophageal varices, for which five bands were deployed during endoscopic variceal ligation (Figure 4). A mosaic mucosal pattern involving the fundus, body, and antrum, consistent with portal hypertensive gastropathy, was also observed (Figure 5).

Initially, the patient demonstrated symptomatic improvement with reduction in abdominal distension and peripheral oedema. Leucocytosis was also reducing with cytoreductive therapy. However, at two-week follow-up, she developed severe neutropenia complicated by sepsis. Despite intensive supportive management, she developed multiorgan dysfunction secondary to neutropenic sepsis and subsequently died.

## Discussion

BCS is an uncommon vascular disorder resulting from hepatic venous outflow obstruction. MPNs, particularly PV, constitute the most common systemic prothrombotic cause, accounting for nearly half of all cases. However, the underlying haematological disorder frequently remains unrecognised because hepatic manifestations often dominate the initial clinical picture.

The diagnosis of MPN-associated BCS is particularly challenging because portal hypertension, splenomegaly, haemodilution, hypersplenism, and iron deficiency may obscure the classical haematological phenotype of PV. Consequently, erythrocytosis or thrombocytosis may be absent at presentation despite an underlying clonal myeloproliferative disorder. This diagnostic pitfall underscores the importance of maintaining a high index of suspicion in patients with unexplained splanchnic venous thrombosis [6].

The identification of a JAK2 V617F mutation remains the most important diagnostic clue in this setting [7]. Approximately 90-95% of patients with PV harbour the JAK2 mutation, and current international recommendations advocate routine molecular screening for JAK2 mutations in all patients presenting with BCS or other unexplained splanchnic vein thromboses, irrespective of peripheral blood counts. Early molecular diagnosis enables prompt institution of disease-specific therapy and appropriate long-term thrombotic risk reduction.

Another noteworthy aspect of this case was the absence of the expected suppression of serum EPO. Although low serum EPO is included among the diagnostic criteria for PV, normal or even elevated levels have occasionally been reported in patients with chronic liver disease and splanchnic venous thrombosis [8]. Hepatic ischemia, altered hepatic metabolism, and chronic hypoxia may influence EPO regulation, limiting its diagnostic utility in this setting. Therefore, serum EPO should be interpreted cautiously and should not dissuade clinicians from pursuing molecular evaluation when clinical suspicion remains high.

Management of MPN-associated BCS requires a multidisciplinary approach targeting both the thrombotic disorder and the complications of portal hypertension. Current management strategies include lifelong anticoagulation, cytoreductive therapy in patients with an underlying MPN, and a stepwise approach to portal hypertension, progressing from medical therapy to endovascular interventions such as transjugular intrahepatic portosystemic shunt (TIPS) and, when necessary, liver transplantation. Early recognition of the underlying MPN is crucial because treatment directed at the clonal haematological disorder reduces the risk of recurrent thrombosis and may improve long-term outcomes.

This case also highlights the potential complications of cytoreductive therapy. Hydroxyurea is generally considered first-line treatment for high-risk PV because of its efficacy and acceptable safety profile; however, severe myelosuppression and neutropenic sepsis, although uncommon, remain recognised adverse effects that necessitate careful monitoring of blood counts, particularly during treatment initiation and dose escalation.

## Conclusions

In conclusion, this case emphasises that BCS may represent the first manifestation of an occult JAK2-positive MPN. Clinicians should consider routine JAK2 mutation testing in all patients with unexplained hepatic or splanchnic venous thrombosis, even in the absence of classical haematological features or with non-suppressed serum EPO levels. Early recognition of the underlying myeloproliferative disorder has important implications for diagnosis, risk stratification, and long-term management.
